# Effects of coconut oil, olive oil, and butter on plasma fatty acids and metabolic risk factors: a randomized trial

**DOI:** 10.1016/j.jlr.2024.100681

**Published:** 2024-10-28

**Authors:** Solomon A. Sowah, Albert Koulman, Stephen J. Sharp, Fumiaki Imamura, Kay-Tee Khaw, Nita G. Forouhi

**Affiliations:** Medical Research Council Epidemiology Unit, University of Cambridge School of Clinical Medicine, Cambridge, UK

**Keywords:** coconut oil, olive oil, butter, saturated fatty acids, randomized trial

## Abstract

There is limited evidence on the effects of different dietary sources of fats on detailed blood fatty acids (FAs). We aimed to evaluate the effects of coconut oil, olive oil and butter on circulating FA concentrations, and examine the associations between changes in plasma FAs and changes in metabolic markers. We conducted secondary analyses in the COB (coconut oil, olive oil and butter) Trial that evaluated 96 healthy adults in a 4-week parallel randomized clinical trial of three dietary interventions: 50 g/d of extra-virgin coconut oil (n = 30), extra-virgin olive oil (n = 33), or unsalted butter (n = 33). We measured plasma phospholipid FA concentrations (mol% of total) using gas chromatography. Using linear regression, we estimated the effects of the interventions on changes in FAs and the associations of changes in selected FAs with changes in metabolic markers. Coconut oil doubled lauric acid (C12:0) and myristic acid (C14:0), butter increased those to a lesser extent, and olive oil reduced those. β (95% confidence interval) for changes in C12:0 comparing coconut oil to butter and olive oil were +0.04 (0.03–0.05) and +0.05 (0.04–0.06) mol%, respectively; for C14:0, +0.24 (0.17–0.32) and +0.37 (0.29–0.45), respectively. Olive oil increased oleic acid (OA) approximately by 1 mol%, while coconut oil and butter had little effect on OA. Butter increased odd-chain SFAs and trans-FAs while olive oil and coconut oil decreased them. Changes in FAs mostly showed no significant associations with changes in metabolic markers. The interventions of equal amounts of different food FA sources altered circulating FA concentrations differently.

Dietary guidelines for cardiometabolic health recommend limiting the consumption of saturated fat by replacing this with unsaturated fat, but there has been increasing interest in the evidence that not all saturated fats have similar metabolic effects. The chain length of individual saturated fatty acids (SFAs) and their formulation within the food matrix likely influences their metabolic actions and subsequent health effects (1). Epidemiological evidence is accumulating showing variation in the association between individual objectively measured SFAs in blood and cardiometabolic disease risk (2, 3, 4). As intermediate factors between the consumption of dietary saturated fat and cardiometabolic disease outcomes, lipid concentrations are useful indicators of the health impact of dietary fats and oils. We previously reported, from the COB Trial, that the consumption of extra-virgin coconut oil, rich in lauric acid (C12:0), a medium-chain SFA, led to significantly decreased low-density lipoprotein cholesterol (LDL-C) and increased high-density lipoprotein cholesterol (HDL-C) as compared with butter consumption, rich in long-chain palmitic (C16:0) and stearic (C18:0) SFAs (5). However, despite the relevance of dietary fat consumption to blood FA concentrations (6) and in turn the impact of blood FAs on disease risk, the relative effects of different sources of dietary fats on individual blood FA concentrations are sparsely reported.

Over the past decades, numerous studies have linked dietary intakes to tissue concentrations of individual fatty acids (FAs). Cross-sectional studies have shown associations between individual plasma FAs and self-reported intake of specific sources of dietary fats (6), although these findings are limited by potential confounding (6, 7) and dietary assessment errors (8, 9). Intervention studies have evaluated the differential effects of specifically formulated fats, natural food sources or specifically prepared meals on individual FAs (6). However, to our knowledge, little evidence is available from research directly comparing the effects of commonly consumed dietary fats on individual FAs that include odd-chain SFAs and *trans* unsaturated FAs. In this study, therefore, we aimed to evaluate the effects of the consumption of equal amounts of extra-virgin coconut oil, extra-virgin olive oil and unsalted butter for four weeks on plasma phospholipid FAs in healthy middle-aged adults in the previously conducted COB randomized trial (5). Our secondary objective was to assess the association between the changes in major FAs and changes in metabolic markers, mainly plasma lipids.

## Patients and methods

### Study design and participants

We conducted a secondary analysis of data from a four-week parallel randomized clinical trial conducted in Cambridge, United Kingdom, over June to July 2017 primarily to evaluate the effects of two sources of SFAs, that is, coconut oil and butter, and a major source of monounsaturated FAs (MUFAs), that is, olive oil, on plasma LDL-C concentrations. Secondary outcomes included plasma concentrations of total cholesterol (TC), triglycerides, HDL-C, TC/HDL-C ratio, and non-HDL-C. Further secondary outcomes were anthropometric measurements i.e., weight, body mass index (BMI) and body fat percentage, systolic and diastolic blood pressure, fasting plasma glucose concentrations and C-reactive protein (CRP) (5).

The original trial design, including power calculations, participants’ eligibility criteria and recruitment procedures, intervention allocation, data collection, and outcome assessments were reported previously (supplemental Fig. S1 for the participant selection) (5). Briefly, participant recruitment was conducted by a British Broadcasting Corporation (BBC) coordinator through the BBC website. A total of 96 participants (%women = 70.8%) were recruited, meeting the eligibility criteria: aged 50–75 years, free of major chronic diseases such as diabetes, cancer or CVD, and not on lipid-lowering medications eg, statins. The participants were randomly allocated to one of three intervention groups for four weeks: 50 g/d extra virgin coconut oil (1.8 MJ) (n = 30), 50 g/d extra virgin olive oil (1.8 MJ) (n = 33) or 50 g/d unsalted butter (1.5 MJ) (n = 33). The FA profiles of these fat sources, which have been reported previously (5), are presented in supplemental Table S1. The coconut oil contained 93.8% saturated fat, predominantly lauric acid (C12:0) and myristic acid (C14:0). In butter, 64.2% was saturated fat, with C16:0, C18:0 and C14:0 as the main FA constituents, and OA the most common MUFA. Olive oil contained 63.5% OA, but also palmitic acid, and contained 11.9% LA. Butter also contained specific FAs such as *trans*-FAs and odd-chain saturated FAs (C15:0; C17:0). Of the 96 randomized participants, two did not attend the baseline assessment, and missing information included five participants without plasma FAs, and eight without metabolic outcomes data, because of loss to follow-up or technical reasons.

During the intervention, participants were advised to maintain their usual diets and to incorporate the provided fats into their usual diets either as a substitute for other fats in their usual diets or as supplements. Participants attended two assessments, one at baseline in June 2017 and the other in July 2017, i.e., after four weeks of the intervention. During each assessment visit to the study center, anthropometric measurements were taken and information on health, and health behaviors including level of physical activity and dietary intake using the online 24-h DietWebQ, was collected. Participants were further asked to report their level of compliance with the intervention by selecting one of six options: 0%, 1%–24%, 25%–49%, 50%–74%, 75%–99%, or 100%. Additionally, participants provided 20 ml of fasting blood sample during each assessment visit. Blood samples were immediately stored at 4°C, before transportation on the same day to the laboratory for further processing, and storage at −80°C for subsequent analysis. Self-reported compliance with the assigned interventions was also assessed after the intervention period. Approval for this study was granted by the University of Cambridge Human Biology Research Ethics Committee (HBREC 2017.05), and all participants provided written informed consent before enrolment. The study was designed and conducted in accordance with the Declaration of Helsinki (10) and was prospectively registered at clinicaltrials.gov (NCT03105947).

### Measurement of plasma phospholipid fatty acid concentrations and concentrations of metabolic markers

Using stored blood samples from the COB trial we measured the concentrations (mol%) of 37 plasma phospholipid FAs, comprising 15 SFAs, eight MUFAs, 11 polyunsaturated FAs (PUFAs), and three *trans-*FAs. This biochemical analysis was conducted at the Medical Research Council Human Nutrition Research, Cambridge, United Kingdom, using previously described automated assay methods developed for FA profiling of the plasma phospholipid fraction (11). In brief, the method involved solvent hydrolysis of phospholipids, followed by methylation of the liberated FAs to yield volatile FA methyl esters. The different FA methyl esters were separated and peak-detected with gas chromatography (7890N, Agilent Technologies) equipped with a split/splitless injector system, a flame ionization detection, and a 30-m-long 0.25-mm-radius capillary column (J&W HP-88, Agilent Technologies). Human and equine plasma samples were used for quality control (Sera Laboratories International). The identification of individual FA methyl esters was performed by comparing their retention times with those of commercial FA methyl ester standards. Each FA was expressed as a percentage of the total FA signal. According to existing biological knowledge, the concentrations of four FA classes were calculated as follows: coconut oil FAs as the sum of C12:0 and C14:0; dairy FAs as the sum of pentadecanoic acid (C15:0), heptadecanoic acid (C17:0), and *trans-*palmitoleic acid (C16:1t); *trans*-FAs as the sum of C16:1t, elaidic acid (C18:1n9t) and C18:2n6t; and MUFAs as the sum of palmitoleic acid (C16:1), oleic acid (C18:1n9c; OA) and heneicosanoic acid (C20:1). The analyses of plasma TC, HDL-C, triglycerides, glucose concentrations, and CRP, and the derivation of LDL-C were performed at the Core Biochemical Assay Laboratory, Cambridge University Hospitals, and details have been described previously (5).

### Statistical analyses

Baseline characteristics were summarized using frequency (percentages) for categorical variables and mean ± standard deviation (SD) for continuous variables. Comparisons between the three intervention groups with respect to each outcome were evaluated based on a *P*-value, calculated using an analysis of covariance model, with change from baseline as the dependent variable, and the intervention group (2 indicator variables) and the baseline value of the dependent variable as independent variables. The increase in *R*^2^ comparing the model with the intervention and that without was calculated. The same model was used to estimate differences and 95% confidence intervals (CI) between each pair of intervention groups, i.e., coconut oil and olive oil, coconut oil and butter, and olive oil and butter. These analyses for our primary aim followed the intention-to-treat principle, whereby all 96 participants were analyzed in the group to which they were randomized, irrespective of their level of adherence to the assigned intervention (12). Thus, the primary analysis incorporated five participants with missing information on FA data. A secondary per-protocol (PP) analysis included participants who reported at least 75% adherence to their assigned intervention.

Multiple linear models were fitted to estimate the association of changes in C12:0, C14:0, C16:0, C18:0, C18:1n9, and linoleic acid (C18:2n6; LA) concentrations with changes in the following metabolic markers: triglycerides, TC, LDL-C, HDL-C, non-HDL-C, the ratio of TC to HDL-C (TC/HDL-C ratio), and CRP. The regression coefficients and 95% CIs represented the difference in the change in each metabolic marker per one SD change in the concentration of each FA. The models were adjusted for age, sex, baseline BMI, baseline value of the outcome (i.e., metabolic marker) and randomization group. As the COB Trial specified LDL-C as the primary outcome and its relative novelty lay in the evaluation of coconut oil, high in 12:0 and 14:0, we particularly highlighted that variable.

To account for the multiple tests for the intervention arms and fatty acid variables, we applied false discovery rate correction to *P*-values (13). All analyses were performed using Stata version 14.2 (StataCorp. LP).

## Results

### Baseline characteristics

The distribution of baseline characteristics was similar between the intervention arms (Table 1). The participants were aged 60 years on average. Two-thirds of the participants were female, and half of the participants were overweight or obese, with mean ± SD BMI of 25.1 ± 4.2 kg/m^2^. The amount of dietary fat consumed as part of the intervention was equivalent to 24.5 ± 12.1% of baseline energy intake for those in the coconut oil group, 24.0 ± 9.2% for the olive oil group, and 17.2 ± 6.1% for the butter group.

**Table 1:** Baseline characteristics of participants in the COB trial according to intervention group^a^

	Coconut oil (n = 29)	Olive oil (n = 33)	Butter (n = 32)
Men, n (%)	11 (37.9)	9 (28.1)	11 (33.3)
Age (years)	59.1 ± 6.1	61.5 ± 5.8	59.1 ± 6.4
Energy intake, EI (MJ)	8.9 ± 3.7	8.2 ± 2.2	9.7 ± 3.1
Carbohydrate intake (% EI)	43.6 ± 8.9	41.4 ± 8.7	42.8 ± 11.6
Fat intake (% EI)	37.3 ± 7.4	36.7 ± 8.7	36.6 ± 10.4
Protein intake (% EI)	14.8 ± 4.4	16.0 ± 3.7	15.8 ± 2.9
Total cholesterol (mmol/l)	5.9 ± 1.0	6.0 ± 0.9	5.9 ± 1.0
HDL-C (mmol/l)	2.0 ± 0.5	1.8 ± 0.5	1.9 ± 0.5
LDL-C (mmol/l)	3.5 ± 0.9	3.7 ± 1.0	3.5 ± 0.9
Total/HDL-C ratio	3.2 ± 0.9	3.5 ± 1.2	3.2 ± 0.8
Glucose (mmol/l)	5.3 ± 0.4	5.4 ± 0.5	5.4 ± 0.5
Weight (kg)	73.9 ± 15.1	71.1 ± 14.5	70.8 ± 11.7
Waist (cm)	85.4 ± 11.9	86.2 ± 11.5	83.7 ± 8.1
Body fat (%)	29.7 ± 10.2	31.5 ± 9.6	29.2 ± 9.0
BMI (kg/m^2^)	25.5 ± 4.5	25.0 ± 4.5	24.8 ± 3.5
Systolic blood pressure (mmHg)	131.4 ± 18.8	133.1 ± 16.5	136.5 ± 18.8
Diastolic blood pressure (mmHg)	79.8 ± 9.3	78.1 ± 6.7	81.0 ± 12.0
Triglycerides (mmol/l)	0.89 (0.74, 1.10)	0.92 (0.70, 1.20)	0.95 (0.80, 1.29)
C-reactive protein (mg/l)	1.04 (0.48, 2.12)	1.08 (0.64, 2.13)	1.13 (0.58, 2.61)

a n = 94. Two individuals did not contribute to the baseline (and follow-up assessment) after randomization. Values are mean ± SD for all continuous variables except triglycerides and CRP for which the median (interquartile range) has been presented. HDL-C = high-density lipoprotein cholesterol, LDL-C = low-density lipoprotein cholesterol.

### Changes in plasma phospholipid fatty acid concentrations after the intervention

At the end of the intervention, almost all participants (98.9%) reported a compliance level of at least 50%. Specifically, compliance levels were 50%–74% in 8.0% of participants, 75%–99% in 27.3% of participants, and 100% in 63.6% of participants. However, one participant (1.1%) reported no compliance (0%). Table 2 shows the descriptive statistics of the 37 plasma FAs and the four FA classes before and after the four-week intervention, and their percentage changes relative to baseline. Table 3 presents effect estimates based on pairwise comparisons between the intervention groups. The interventions caused changes in concentrations of several FAs. For example, the interventions influenced odd-chain SFAs (C15:0, C17:0 and C23:0; increase in *R*^2^ due to intervention 0.21 to 0.33), coconut oil FAs (C12:0, C14:0; ΔR^2^ = 0.51 and 0.45, respectively), OA (ΔR^2^ = 0.22), and *trans*-LA (*trans*-C18:2n6; ΔR^2^ = 0.19), but not palmitic acid (C16:0; ΔR^2^ = 0.03) and LA (*cis*-C18:2n6; ΔR^2^ = 0.06).

**Table 2:** Plasma phospholipid fatty acid concentrations (mol%) at baseline, week 4 and changes from baseline to week 4 in the COB trial, according to the intervention group^a^

	Coconut oil (n = 30)			Olive oil (n = 33)			Butter (n = 33)		
	Baseline	Week 4	Relative change, %	Baseline	Week 4	Relative change, %	Baseline	Week 4	Relative change, %
Saturated FAs									
Caprylic acid, C8:0	0.01 ± 0.02	0.01 ± 0.02	17.6 ± 67.3	0.01 ± 0.02	0.00 ± 0.01	26.9 ± 153.1	0.01 ± 0.01	0.01 ± 0.02	11.5 ± 97.0
Capric acid, C10:0	0.01 ± 0.02	0.01 ± 0.02	−3.2 ± 44.5	0.01 ± 0.01	0.01 ± 0.01	−29.1 ± 59.4	0.01 ± 0.02	0.01 ± 0.02	29.7 ± 144
Undecylic acid, C11:0	0.01 ± 0.01	0.01 ± 0.01	−27.0 ± 59.6	0.00 ± 0.01	0.01 ± 0.01	−34.8 ± 43.8	0.00 ± 0.01	0.01 ± 0.01	−10.3 ± 92.1
Lauric acid, C12:0^b^	0.03 ± 0.01	0.07 ± 0.03	204 ± 155	0.02 ± 0.01	0.02 ± 0.01	0.5 ± 43.8	0.02 ± 0.01	0.03 ± 0.02	22.6 ± 86.9
Tridecylic acid, C13:0	0.01 ± 0.02	0.01 ± 0.02	−14.7 ± 85.9	0.01 ± 0.03	0.01 ± 0.03	−19.2 ± 48.1	0.00 ± 0.01	0.00 ± 0.01	−6.1 ± 117
Myristic acid, C14:0^b^	0.40 ± 0.13	0.72 ± 0.20	97.0 ± 90.7	0.43 ± 0.09	0.36 ± 0.08	−14.9 ± 15.0	0.38 ± 0.10	0.47 ± 0.15	26.6 ± 44.0
Pentadecanoic acid, C15:0^b^	0.23 ± 0.05	0.21 ± 0.05	−6.7 ± 17.8	0.24 ± 0.05	0.21 ± 0.04	−10.7 ± 19.8	0.23 ± 0.04	0.27 ± 0.04	18.1 ± 20.5
Palmitic acid, C16:0	29.7 ± 1.67	29.8 ± 1.24	0.2 ± 3.9	29.8 ± 1.32	29.4 ± 1.20	−1.4 ± 3.2	30.0 ± 1.39	29.6 ± 1.38	−1.4 ± 3.1
Heptadecanoic acid, C17:0^b^	0.61 ± 0.18	0.53 ± 0.18	−11.7 ± 14.2	0.53 ± 0.15	0.51 ± 0.16	−3.6 ± 12.4	0.53 ± 0.19	0.56 ± 0.18	7.2 ± 11.1
Stearic acid, C18:0	14.0 ± 0.96	14.1 ± 0.96	0.8 ± 6.3	13.95 ± 0.93	13.82 ± 0.91	−0.4 ± 3.8	13.9 ± 0.89	14.20 ± 0.80	2.4 ± 4.4
Arachidic acid, C20:0	0.15 ± 0.02	0.15 ± 0.02	2.1 ± 11.6	0.14 ± 0.02	0.15 ± 0.02	8.2 ± 14.3	0.14 ± 0.02	0.13 ± 0.02	−1.6 ± 13.1
Heneicosanoic acid, C21:0	0.01 ± 0.02	0.00 ± 0.01	−84.1 ± 39.0	0.01 ± 0.02	0.01 ± 0.02	−13.4 ± 64.0	0.01 ± 0.02	0.01 ± 0.03	−45.4 ± 109
Behenic acid, C22:0	0.21 ± 0.03	0.21 ± 0.04	−1.7 ± 15.4	0.22 ± 0.04	0.22 ± 0.04	0.9 ± 8.4	0.21 ± 0.03	0.21 ± 0.04	0.3 ± 13.3
Tricosanoic acid, C23:0^b^	0.11 ± 0.02	0.10 ± 0.02	−3.7 ± 14.8	0.11 ± 0.02	0.12 ± 0.02	3.9 ± 11.3	0.11 ± 0.02	0.12 ± 0.03	16.4 ± 21.0
Lignoceric acid, C24:0	0.22 ± 0.03	0.23 ± 0.04	5.0 ± 18.6	0.24 ± 0.03	0.24 ± 0.03	1.0 ± 8.8	0.23 ± 0.04	0.23 ± 0.05	1.6 ± 17.0
PUFAs									
α-Linolenic acid, C18:3n3^b^	0.35 ± 0.16	0.39 ± 0.11	19.3 ± 38.7	0.39 ± 0.13	0.31 ± 0.08	−15.0 ± 19.5	0.32 ± 0.10	0.38 ± 0.14	19.1 ± 32.0
Eicosapentaenoic acid, C20:5n3	1.79 ± 1.29	1.77 ± 0.96	11.0 ± 57.5	1.62 ± 0.76	1.49 ± 1.12	−4.9 ± 37.3	1.50 ± 0.67	1.81 ± 1.04	26.3 ± 51.0
Docosapentaenoic acid, C22:5n3^b^	0.99 ± 0.14	0.93 ± 0.14	−5.6 ± 14.3	1.02 ± 0.20	0.90 ± 0.16	−10.5 ± 11.4	0.96 ± 0.15	1.00 ± 0.17	4.2 ± 14.3
Docosahexaenoic acid C22:6n3	4.25 ± 1.16	3.65 ± 0.79	−11.3 ± 17.9	4.06 ± 1.42	3.72 ± 1.15	−8.2 ± 13.7	4.00 ± 0.97	3.92 ± 1.29	−2.9 ± 17.3
Linoleic acid, C18:2n6c	23.2 ± 3.07	24.2 ± 2.73	5.6 ± 10.5	22.9 ± 3.14	23.4 ± 2.85	2.7 ± 8.6	22.7 ± 2.37	22.7 ± 2.96	0.3 ± 10.3
γ-Linolenic acid, C18:3n6	0.07 ± 0.03	0.09 ± 0.04	43.8 ± 107	0.09 ± 0.04	0.09 ± 0.03	2.3 ± 39.2	0.09 ± 0.04	0.09 ± 0.03	−1.8 ± 34.4
Eicosadienoic acid, C20:2n6^b^	0.33 ± 0.06	0.35 ± 0.06	10.5 ± 21.7	0.32 ± 0.04	0.31 ± 0.04	−2.9 ± 10.3	0.32 ± 0.05	0.32 ± 0.04	3.7 ± 14.0
Dihomo-γ-linolenic acid, C20:3n6	2.58 ± 0.81	2.74 ± 0.79	8.3 ± 14.8	2.77 ± 0.49	2.81 ± 0.52	3.2 ± 17.3	2.85 ± 0.63	2.82 ± 0.66	1.1 ± 19.6
Arachidonic acid, C20:4n6	8.70 ± 1.88	7.91 ± 1.76	−7.3 ± 11.7	8.67 ± 1.76	8.42 ± 1.65	−3.1 ± 10.0	8.81 ± 1.60	8.41 ± 1.33	−2.7 ± 12.2
Docosatetraenoic acid, C22:4n6	0.24 ± 0.07	0.23 ± 0.07	−3.9 ± 12.3	0.26 ± 0.06	0.24 ± 0.07	−4.6 ± 21.0	0.25 ± 0.06	0.24 ± 0.06	−1.3 ± 14.1
Docosapentenoic acid, C22:5n6	0.14 ± 0.06	0.13 ± 0.06	−9.0 ± 13.5	0.12 ± 0.09	0.11 ± 0.09	−2.1 ± 19.5	0.17 ± 0.06	0.17 ± 0.07	3.3 ± 19.8
MUFAs									
Myristoleic acid, C14:1	0.02 ± 0.01	0.02 ± 0.01	3.0 ± 64.5	0.02 ± 0.01	0.02 ± 0.02	2.3 ± 137.2	0.02 ± 0.01	0.03 ± 0.01	46.2 ± 61.1
Pentadecenoic acid, C15:1	0.03 ± 0.04	0.02 ± 0.02	−46.9 ± 56.8	0.02 ± 0.02	0.02 ± 0.03	−22.9 ± 60.4	0.02 ± 0.02	0.02 ± 0.02	−16.1 ± 64.2
Palmitoleic acid, C16:1c	0.50 ± 0.16	0.53 ± 0.16	6.7 ± 20.8	0.53 ± 0.19	0.50 ± 0.15	−3.0 ± 21.8	0.53 ± 0.19	0.51 ± 0.17	−1.2 ± 22.0
Heptadecenoic acid, C17:1	0.05 ± 0.02	0.05 ± 0.03	−3.7 ± 34.7	0.04 ± 0.02	0.05 ± 0.02	22.5 ± 80.7	0.05 ± 0.03	0.07 ± 0.02	42.9 ± 95.9
Oleic acid, C18:1n9c^b^	10.1 ± 1.11	9.89 ± 1.11	−1.4 ± 13.2	10.5 ± 1.29	11.6 ± 1.09	12.9 ± 14.4	10.7 ± 1.14	10.8 ± 1.04	0.5 ± 12.7
Gondoic acid, C20:1n9	0.25 ± 0.08	0.24 ± 0.07	1.2 ± 30.5	0.26 ± 0.07	0.25 ± 0.05	−3.4 ± 19.3	0.24 ± 0.05	0.24 ± 0.10	1.8 ± 32.6
Erucic acid, C22:1n9	0.09 ± 0.02	0.08 ± 0.02	5.3 ± 32.5	0.09 ± 0.02	0.09 ± 0.02	4.1 ± 27.6	0.08 ± 0.02	0.08 ± 0.02	−3.6 ± 30.0
Nervonic acid, C24:1	0.37 ± 0.05	0.36 ± 0.06	−3.0 ± 14.8	0.39 ± 0.07	0.37 ± 0.07	−3.4 ± 10.5	0.36 ± 0.07	0.33 ± 0.06	−5.5 ± 16.8
*trans*-FAs									
Transpalmitoleic acid, C16:1t	0.06 ± 0.03	0.05 ± 0.03	−9.6 ± 41.7	0.05 ± 0.03	0.04 ± 0.02	1.3 ± 88.1	0.04 ± 0.02	0.06 ± 0.03	55.4 ± 139.3
Elaidic acid, C18:1n9t	0.12 ± 0.09	0.10 ± 0.03	0.7 ± 48.9	0.14 ± 0.11	0.10 ± 0.06	−12.5 ± 33.9	0.09 ± 0.03	0.11 ± 0.04	19.1 ± 23.4
*Trans* linoleic acid, C18:2n6t^b^	0.07 ± 0.02	0.06 ± 0.01	0.3 ± 25.8	0.07 ± 0.01	0.06 ± 0.01	−4.6 ± 15.8	0.07 ± 0.02	0.08 ± 0.02	16.9 ± 19.6
Fatty acid classes									
Coconut FAs^b^	0.43 ± 0.14	0.79 ± 0.23	102 ± 90.3	0.45 ± 0.09	0.38 ± 0.09	−14.3 ± 15.7	0.41 ± 0.11	0.50 ± 0.17	26.2 ± 45.3
Dairy FAs^b^	0.90 ± 0.22	0.80 ± 0.22	−10.9 ± 15.0	0.81 ± 0.18	0.76 ± 0.19	−6.2 ± 14.0	0.80 ± 0.22	0.89 ± 0.22	11.0 ± 11.8
Total *trans*-FAs	0.24 ± 0.10	0.21 ± 0.05	−6.0 ± 32.3	0.25 ± 0.12	0.20 ± 0.06	−11.7 ± 28.2	0.20 ± 0.04	0.25 ± 0.07	20.8 ± 23.6
MUFAs^b^	10.80 ± 1.19	10.66 ± 1.16	−1.2 ± 12.6	11.27 ± 1.41	12.35 ± 1.13	11.6 ± 13.8	11.47 ± 1.17	11.53 ± 1.11	0.3 ± 12.2

a n = 91 after excluding participants with missing data on fatty acids. Values are means ± SD. Coconut oil fatty acids = C12:0 + C14:0; Dairy fatty acids = C15:0 + C17:0 + C16:1t; Trans fatty acids = C16:1t + C18:1n9t + C18:2n6t; MUFAs = C16:1 + C18:1 + C20:1.

b Statistically significant effect comparing intervention groups, after correction for false discovery rate (*P* < 0.002) upon the multiple tests for the intervention arms and fatty acid variables.

**Table 3:** Pairwise intervention group comparisons of changes in mol% of plasma phospholipid fatty acids in the COB trial (n = 96)^a^

Fatty Acid Types		Effect Estimate (95% Confidence interval)^a^			ΔR^c^
	Mean change ± SD^b^	Coconut oil versus Olive oil	Coconut oil versus Butter	Butter versus Olive oil	
Saturated FAs					
Lauric acid, C12:0^d^	0.01 ± 0.03	0.05 (0.04–0.06)^a^	0.04 (0.03–0.05)^a^	0.00 (−0.01 to 0.01)	0.51
Myristic acid, C14:0^d^	0.10 ± 0.22	0.37 (0.29–0.45)^a^	0.24 (0.17–0.32)^a^	0.13 (0.05–0.20)^a^	0.45
Pentadecanoic acid, C15:0	0.00 ± 0.05	0.01 (−0.01 to 0.03)	−0.06 (−0.08 to −0.04)^a^	0.07 (0.05–0.08)^a^	0.33
Palmitic acid, C16:0^d^	−0.30 ± 1.04	0.43 (−0.04 to 0.90)	0.35 (−0.11 to 0.82)	0.08 (−0.37 to 0.53)	0.03
Heptadecanoic acid, C17:0	−0.02 ± 0.08	−0.04 (−0.08 to −0.01)	−0.09 (−0.13 to −0.06)^a^	0.05 (0.01–0.08)	0.22
Stearic acid, C18:0^d^	0.12 ± 0.64	0.18 (−0.13 to 0.48)	−0.21 (−0.51 to 0.10)	0.38 (0.09–0.67)	0.06
Arachidic acid, C20:0	0.00 ± 0.02	−0.01 (−0.01 to 0.00)	0.01 (0.00–0.02)	−0.01 (−0.02 to −0.01)	0.10
Behenic acid, C22:0	0.00 ± 0.03	−0.01 (−0.02 to 0.00)	0.00 (−0.02 to 0.01)	0.00 (−0.02 to 0.01)	0.02
Tricosanoic acid, C23:0	0.01 ± 0.02	−0.01 (−0.02 to 0.00)	−0.02 (−0.03 to −0.01)^a^	0.01 (0.00–0.02)	0.21
Lignoceric acid, C24:0	0.00 ± 0.03	0.00 (−0.01 to 0.02)	0.01 (−0.01 to 0.02)	0.00 (−0.02 to 0.01)	0.00
Polyunsaturated FAs					
α-Linolenic acid, C18:3n3	0.01 ± 0.11	0.09 (0.04–0.14)^a^	0.00 (−0.05 to 0.04)	0.10 (0.05–0.14)^a^	0.15
Eicosapentaenoic acid, C20:5n3	0.04 ± 0.99	0.15 (−0.33 to 0.62)	−0.24 (−0.71 to 0.23)	0.39 (−0.06 to 0.84)	0.03
Docosapentaenoic acid, C22:5n3	−0.05 ± 0.14	0.05 (−0.02 to 0.11)	−0.09 (−0.15 to −0.02)	0.13 (0.07–0.19)	0.15
Docosahexaenoic acid, C22:6n3	−0.34 ± 0.76	−0.16 (−0.52 to 0.19)	−0.45 (−0.81 to −0.10)	0.29 (−0.05 to 0.63)	0.06
Linoleic acid, C18:2n6^d^	0.50 ± 2.17	0.70 (−0.35 to 1.76)	1.27 (0.23–2.31)	−0.57 (−1.57 to 0.43)	0.06
γ-Linolenic acid, C18:3n6	0.00 ± 0.04	0.01 (−0.01 to 0.03)	0.01 (−0.01 to 0.03)	0.00 (−0.02 to 0.01)	0.02
Eicosadienoic acid, C20:2n6	0.01 ± 0.05	0.04 (0.02–0.06)^a^	0.02 (0.00–0.04)	0.02 (0.00–0.03)	0.12
Dihomo-γ-linolenic acid, C20:3n6	0.06 ± 0.44	0.06 (−0.16 to 0.28)	0.11 (−0.12 to 0.33)	−0.05 (−0.26 to 0.16)	0.01
Arachidonic acid, C20:4n6	−0.43 ± 1.03	−0.39 (−0.89 to 0.11)	−0.38 (−0.87 to 0.11)	−0.01 (−0.48 to 0.46)	0.03
Docosatetraenoic acid, C22:4n6	−0.01 ± 0.04	0.00 (−0.02 to 0.02)	0.00 (−0.02 to 0.01)	0.01 (−0.01 to 0.03)	0.01
Docosapentenoic acid, C22:5n6	0.00 ± 0.03	−0.01 (−0.02 to 0.01)	−0.02 (−0.03 to 0.00)	0.01 (−0.01 to 0.02)^a^	0.07
Monounsaturated FAs					
Myristoleic acid, C14:1	0.00 ± 0.02	0.00 (−0.01 to 0.01)	−0.01 (−0.02 to 0.00)	0.01 (0.00–0.02)	0.05
Pentadecenoic acid C15:1	0.00 ± 0.03	−0.01 (−0.02 to 0.01)	0.00 (−0.01 to 0.01)	0.00 (−0.01 to 0.01)	0.01
Palmitoleic acid, C16:1c	−0.01 ± 0.11	0.04 (−0.01 to 0.09)	0.03 (−0.02 to 0.08)	0.01 (−0.03 to 0.06)	0.03
Heptadecenoic acid, C17:1	0.01 ± 0.03	0.00 (−0.02 to 0.01)	−0.01 (−0.02 to 0.00)	0.01 (0.00–0.02)	0.05
Oleic acid, C18:1n9c^d^	0.34 ± 1.44	−1.67 (−2.21 to −1.12)^a^	−0.71 (−1.26 to −0.16)	−0.96 (−1.48 to −0.44)^a^	0.22
Gondoic acid, C20:1n9	−0.01 ± 0.06	0.00 (−0.03 to 0.03)	−0.01 (−0.05 to 0.02)	0.02 (−0.02 to 0.05)	0.01
Erucic acid, C22:1n9	0.00 ± 0.02	0.00 (−0.01 to 0.01)	0.01 (0.00–0.02)	−0.01 (−0.02 to 0.00)	0.02
Nervonic acid, C24:1	−0.02 ± 0.05	0.00 (−0.03 to 0.02)	0.02 (−0.01 to 0.04)	−0.02 (−0.05 to 0.00)	0.03
*Trans-*FAs					
*Trans-*palmitoleic acid, C16:1t	0.00 ± 0.03	0.00 (−0.01 to 0.02)	−0.02 (−0.03 to 0.00)	0.02 (0.01–0.03)^a^	0.10
Elaidic acid, C18:1n9t	−0.01 ± 0.09	−0.01 (−0.03 to 0.02)	−0.02 (−0.04 to 0.00)	0.01 (−0.01 to 0.04)	0.01
*Trans-*linoleic acid, C18:2n6t	0.00 ± 0.01	0.00 (0.00–0.01)	−0.01 (−0.02 to −0.01)^a^	0.01 (0.01–0.02)^a^	0.19
Fatty acid classes					
Coconut FAs	0.12 ± 0.25	0.42 (0.33–0.50)^a^	0.29 (0.20–0.37)^a^	0.13 (0.05–0.21)	0.47
Dairy FAs	−0.01 ± 0.15	−0.03 (−0.09 to 0.02)	−0.17 (−0.23 to −0.11)^a^	0.13 (0.08–0.19)^a^	0.23
*trans*-FAs	−0.01 ± 0.11	0.01 (−0.03 to 0.04)	−0.04 (−0.08 to −0.01)	0.05 (0.02–0.08)^a^	0.05
MUFAs	0.32 ± 1.47	−1.64 (−2.20 to −1.07)^a^	−0.69 (−1.26 to −0.12)	−0.95 (−1.48 to −0.41)^a^	0.20

a Values are baseline-adjusted differences between each pair of intervention groups in the changes from baseline (95% confidence intervals) (not presenting C8:0, C10:0, C11:0, C13:0 and C21:0 as in Table 2). The superscript was assigned to statistically significant effect estimates after correction for false discovery rate (*P* < 0.002) upon the multiple tests for the intervention arms and fatty acid variables. Coconut oil fatty acids= (C12:0 + C14:0); dairy fatty acids= (C15:0 + C17:0 + C16:1t); trans-fatty acids= (C16:1t + C18:1n9t + C18:2n6t); MUFAs = (C16:1 + C18:1 + C20:1).

b Values are the mean ± SD changes in the concentrations of the plasma fatty acids from baseline to follow-up.

c Increase in R-squared due to inclusion of intervention in the regression analysis adjusting for baseline values of a fatty acid variable.

d Fatty acid variables carried forward to the subsequent analyses for associations with metabolic markers (lipids and C-reactive protein).

C12:0 and C14:0, SFAs abundant in coconut oil (>60% of total FAs) (supplemental Table S1), were present in a relatively low amount of <1.0 mol% in plasma phospholipids on average. Those concentrations doubled in the coconut oil group, increased by 20% in butter, and changed little in the olive oil group. For example, βs (95% confidence intervals, CI) for changes in C12:0 (mean change ±SD = 0.01 ± 0.03 mol%) by comparing coconut oil to butter and olive oil were +0.04 (0.03, 0.05) and +0.05 (0.04, 0.06) mol%, respectively; and for changes in C14:0 (0.10 ± 0.22 mol%) were +0.24 (0.17, 0.32) and +0.37 (0.29, 0.45) mol%, respectively.

Butter increased the concentration of the odd-chain SFAs (C15:0 and C17:0) and total *trans*-FAs, whereas coconut oil and olive oil decreased the concentrations of these FAs. When comparing butter to coconut oil and olive oil, βs (95% CI) in mol% for changes in C15:0 were +0.06 (0.04, 0.08) and +0.07 (0.05, 0.08), respectively; for C17:0, these were +0.09 (0.06, 0.13) and +0.05 (0.01, 0.08); and for changes in total *trans*-FAs, +0.04 (0.01, 0.08) and +0.05 (0.02, 0.08). *Trans*-LA changed by < 0.02 mol%, while the variability of the changes partly depended on the interventions (ΔR^2^ = 0.19): *trans*-LA was increased by butter and decreased by coconut oil and olive oil (Table 3).

Palmitic acid (C16:0) and stearic acid (C18:0), the most abundant fatty acids in butter, were the predominant FAs in plasma phospholipids, making up 40%–45% of total FAs, but the interventions had minimal effect on their concentrations in plasma phospholipids. The effect estimates were minimal, except for the estimate comparing butter to olive oil for changes in stearic acid (C18:0): β (95% CI) = +0.38 (0.09, 0.67) mol%.

Linoleic acid (LA; C18:2n6), the most abundant dietary PUFA, was the most prevalent FA in olive oil among the fat sources, but its blood concentrations were elevated only in the coconut oil group approximately by 1 mol% but little in the other intervention groups (<0.5 mol%). For LA changes, coconut oil compared to butter and olive oil showed β (95% CI) of +0.70 (−0.35, 1.76) and +1.27 (0.23, 2.31) mol%. Among omega-3 FAs, ALA (C18:3n3) was elevated after butter and coconut oil consumption but decreased after olive oil consumption (Table 3). A similar trend was observed for changes in eicosapentaenoic acid (C20:5n3). Plasma phospholipid OA (C18:1n9) increased by 1 mol% or relatively by 12.9% in the olive oil group, changed little in the butter group, and decreased in the coconut oil group (+0.5% and −1.4%, respectively) (Table 3).

### Associations between plasma phospholipid FAs and metabolic markers

**Fig. 1:**
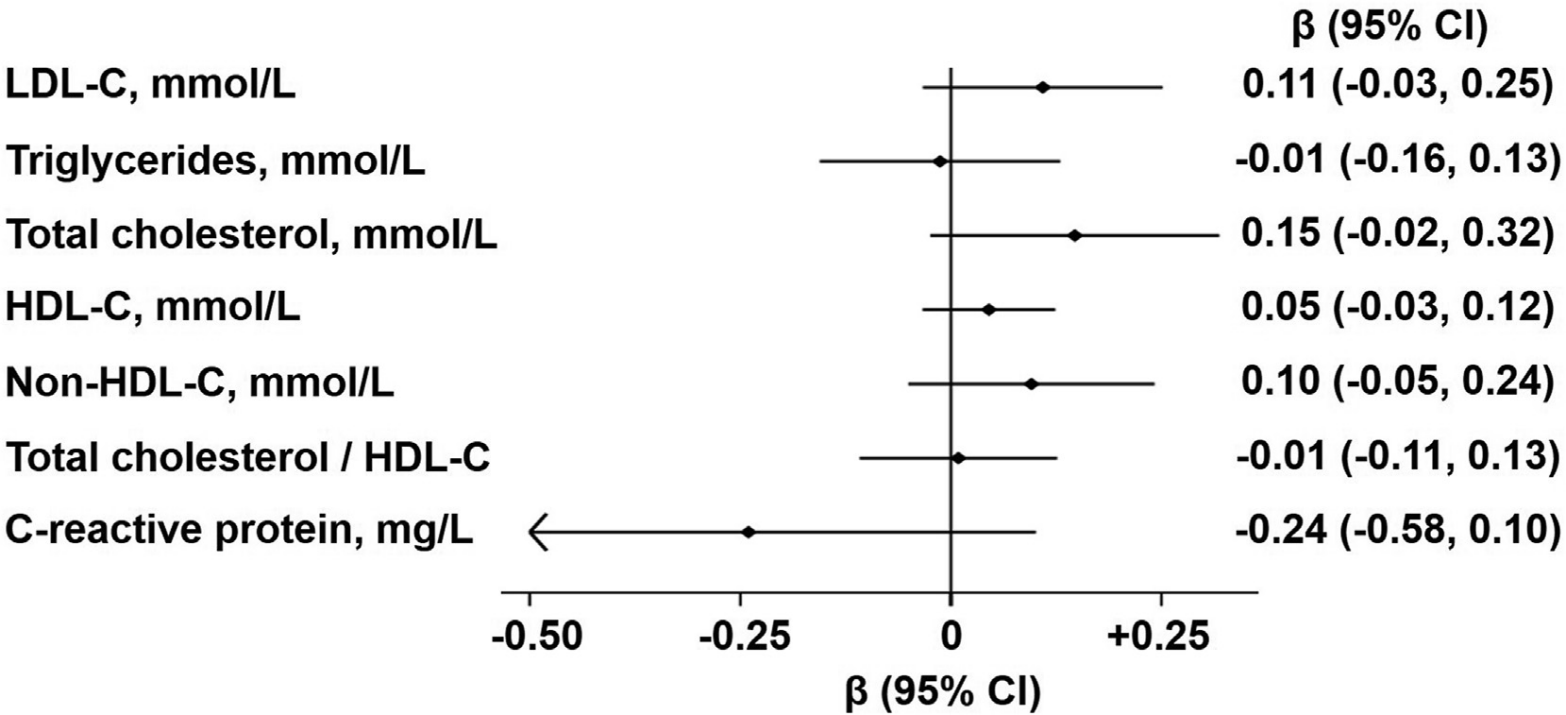
Multivariable-adjusted associations of changes in coconut oil fatty acids (C12:0 + C14:0) with changes in metabolic markers in the COB Trial. N = 88. The regression coefficients and 95% CIs represent the difference in the change in each metabolic marker per one SD change in the concentration of coconut oil fatty acids (C12:0 + C14:0). Models were adjusted for age, sex, baseline body mass index, baseline outcome values and randomization arms. COB, coconut oil, olive oil and butter; HDL-C, high-density lipoprotein cholesterol; LDL-C, low-density lipoprotein cholesterol; SD, standard deviation.

**Fig. 2:**
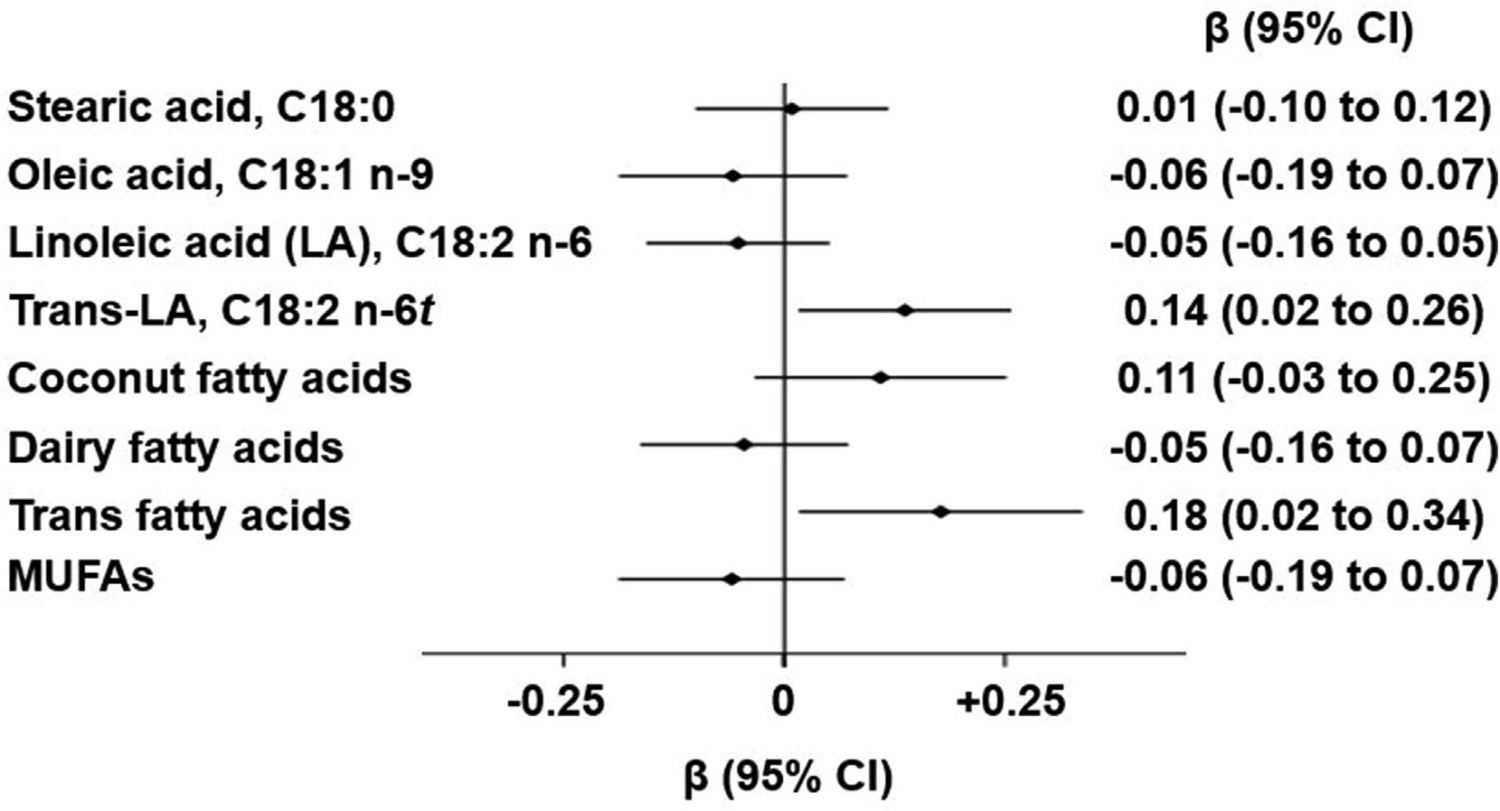
Multivariable-adjusted associations of changes in selected fatty acid variables with changes in low-density lipoprotein cholesterol concentrations in the COB Trial. N = 88. The regression coefficients and 95% CIs represent the difference in the change in LDL-cholesterol (mmol/L) per one SD change in the concentration of each FA. Models were adjusted for age, sex, baseline body mass index, baseline outcome values, and randomization arms. COB, coconut oil, olive oil and butter; coconut oil fatty acids, C12:0 + C14:0; dairy fatty acids, C15:0 + C17:0 + C16:1*t*; *trans*-fatty acids, C16:1*t* + C18:1n9*t* + C18:2n6*t*, monounsaturated fatty acids (MUFAs), C16:1+ C18:1 + C20:1; SD, standard deviation. Δ LDL-C = 0.07 ± 0.49 mmol/L.

The associations between changes in selected plasma phospholipid FAs and changes in metabolic markers during follow-up are shown in Figures 1, 2 and supplemental Table S2. Changes in coconut oil FA class (C12:0+C14:0) showed overall non-significant associations with the metabolic markers (Fig. 1). The other associations of FA changes with changes in metabolic markers had wide confidence intervals or estimates close to the null (Fig. 2, supplemental Table S2). Notably, changes in OA and total MUFAs showed negative associations with changes in HDL-C and positive associations with changes in TC/HDL-C ratio and triglycerides. Changes in trans-LA and total trans-FAs showed significant positive associations with changes in LDL-C, while trans-LA specifically was also associated with changes in non-HDL-C concentrations (Fig. 2 and supplemental Table S2).

## Discussion

In this four-week randomized trial of healthy middle-aged adults, daily consumption of 50 g of coconut oil increased circulating C12:0 on average compared to either 50 g olive oil or 50 g butter. Furthermore, circulating C14:0 was increased by butter, and to a greater extent by coconut oil, but its concentration was decreased by olive oil. The concentration of circulating OA increased after olive oil consumption as would be expected, but decreased or was unchanged after coconut oil or butter consumption. Butter consumption increased the concentrations of individual plasma odd-chain FA (C15:0 and C17:0), the fatty acid groups total *trans*-FAs, and dairy FAs, whereas the concentrations of these FAs were decreased by both coconut oil and olive oil. For metabolic markers, there was no strong evidence for the potential harm of the coconut oil SFAs, i.e., C12:0 and C14:0: changes in concentrations of these SFAs indicated overall null associations with changes in LDL-C, HDL-C and total cholesterol. None of the major FA classes showed clear associations with changes in LDL-C, the primary outcome of this trial, with the exception of the positive associations between changes in *trans*-FAs and changes in LDL-C. Overall, these findings suggest that there are variations in the effects of different dietary fats on individual plasma FAs, partly attributable to dietary FA compositional differences. Moreover, the association between major plasma FAs and biomarkers of lipid dysregulation may differ considerably, including between different types of plasma SFAs.

Lauric (C12:0) and myristic (C14:0) are the main FA components of coconut oil, thus, an increase in their blood concentrations with coconut oil would be expected. In a small supplementation trial among patients with dyslipidemia, plasma triacylglycerol C12:0 was reported to be significantly increased with coconut oil compared to butter (6), consistent with our study. Likewise, the differential effects on C18:0 may be explained by the differences in dietary C18:0 content between the dietary fats. Higher plasma C18:0 has been reported after butter intake in comparison to coconut oil (6) and olive oil (14), although the former was not observed in this current study. Furthermore, despite the higher content of C16:0 in butter relative to both coconut oil and olive oil, no evidence of a differential effect on C16:0 was observed, in line with findings from previous studies (6, 15). The absence of a differential effect on C16:0 may be related to the fact that endogenously-produced C16:0, the major product of de novo hepatic lipogenesis, influenced by alcohol and carbohydrate intake, may have offset potential dietary fat-induced changes in C16:0 (16, 17). Given the accumulated data regarding a higher risk of CVD with higher plasma C16:0 (18, 19), further studies are needed to establish whether C16:0 concentration is less dependent on the C16:0 content of dietary fats.

The increase in the concentrations of odd-chain SFAs (C15:0 and C17:0) with butter and their decreases with both coconut oil and olive oil are consistent with evidence that biomarker concentrations of these FAs were correlated with dairy fat consumption (20, 21). Odd-chain SFAs are present in very low concentrations in the circulation (22), nonetheless, their concentrations have been shown to reflect total dairy fat intake in a few trials and observational studies as discussed previously (4). Our finding was also supportive of the summary evidence that odd-chain SFA concentrations and butter consumption were associated with a lower risk of T2D (3, 4, 23). Findings for CVD incidence or mortality were inconsistent. Odd-chain SFA concentrations were associated with lower CVD incidence (24), whereas butter consumption showed weak positive associations with total mortality and CVD incidence (23). Overall, the available evidence supports the notion that individual SFAs have differential associations with cardiometabolic risks. On the other hand, there are established causal effects of SFAs (mainly even-chain SFAs) on LDL-C, and of LDL-C on major CVDs, while substitution of SFAs for PUFAs or MUFAs is associated with a lower risk of cardiometabolic diseases (25, 26). Therefore, coconut oil and butter are generally considered less healthy (27). The effects of individual SFAs on overall health and individual disease outcomes, therefore, deserve further investigations.

The increase in *trans*-palmitoleic acid and total *trans*-FAs after consumption of butter, but not coconut oil or olive oil, was identified, supporting evidence that these FAs could be obtained from dairy and other ruminant products, but not unprocessed plant oils (28, 29, 30). *Trans*-palmitoleic acid has been considered as a ruminant *trans*-FA, and its plasma concentration is inversely associated with CVD risk factors (31) and the risk of T2D (4), but not ischemic heart disease (32). To our knowledge, associations of blood 18-carbon *trans*-FA with cardiometabolic risks were inconsistent (32, 33). Nonetheless, our finding relating butter consumption to plasma phospholipid 18-carbon *trans*-FAs underpins the caution that blood 18-carbon *trans*-FAs do not necessarily reflect industrially processed sources of *trans*-FAs (eg, hydrogenated oil) (30, 34).

The greater increase in the concentrations of OA after olive oil (comprising ∼64% OA) consumption compared to butter (∼22% OA), may be attributable to the higher content of OA in the former and agrees with increases in OA after olive oil consumption in short-term intervention studies (14, 35). Plasma concentrations of PUFAs, particularly LA, are inversely associated with cardiometabolic disease risk (36, 37). LA is the most predominant n-6 PUFA and is obtained exclusively through dietary intake (38). Thus, the main reason for the absence of differences in LA concentrations after the intervention may be related to the relatively lower content of LA in olive oil compared to major dietary sources of LA such as vegetable oils (39, 40).

Concerning metabolic markers, our findings were overall null for an association between changes in predominantly medium-chain coconut oil FAs (C12:0 and C14:0), and changes in LDL-C, HDL-C, non-HDL-C and TC, which are in line with suggestions that these FAs do not raise blood cholesterol as much as long-chain SFAs such as C16:0 (41, 42). For *trans*-LA, we observed that changes in this FA were positively associated with changes in lipid biomarkers that indicated worse metabolic outcomes (raised LDL-c and TC/HDL-C). *Trans*-LA has different isomeric forms, and they likely have divergent associations with markers of lipid metabolism (43), which was not evaluated in our study. Additionally, the finding of *trans*-LA is at variance with the speculated beneficial metabolic effects of several plasma ruminant *trans*-FAs (4, 43), confirming the need for understanding possible distinct effects of individual *trans*-FAs. The positive association between the changes in total *trans*-FA and changes in LDL-C and TC is consistent with the reported effect of *trans*-FAs from industrial sources (44). As our trial had no intervention of industrially processed fat sources, the finding indicates that *trans*-FAs may exert the cholesterol-raising effect, regardless of the fat source, while confounding by other metabolic factors, such as changes in body fat, might also occur.

The strengths of this study include a randomized design, high compliance, a comprehensive profiling of plasma phospholipid FAs, and an analysis of the FA content of the dietary fats consumed. The free provision of the exact amounts the dietary fats to participants in all the intervention groups reduced the possibility of differential treatment and potential errors due to inaccurate assessment of self-reported dietary fat intake. There are potential limitations of our study. Plasma FAs were assessed as relative concentrations in this study rather than absolute concentrations, whereas relative concentrations have been argued to be useful in evaluating the metabolic state relevant to FA exposure (45) and demonstrated to reflect dietary intakes (6). Our FA assays had a weak resolution for stereoisomeric FA types, being unable to distinguish isomers of LA, for example, including conjugated LAs, or isomers of *trans*-18:1 FAs (*trans*-18:1n9 and *trans*-18:1n7) (46), which may have further differential health effects. Our assays were conducted only twice at baseline and endpoint, and the dose of each fat source was fixed to 50 g/day. Therefore, by design, we could not evaluate dose-response effects regarding duration and volume. The secondary analysis relating changes in plasma FAs to changes in metabolic markers may have been confounded due to metabolic factors correlated with changes in both FAs and the outcome measures. Additionally, changes in plasma FAs were secondary outcomes of the COB trial, while the original sample size/power calculation was performed for the primary outcome (change in LDL cholesterol). However, having collected the data there is still value in reporting estimates of the intervention effects and confidence intervals for relevant secondary outcomes such as these. Lastly, study participants were blinded to the outcome assessment, but not blinded to the dietary intervention. Therefore, we were unable to account for possible differential changes in participants’ behaviors in response to each intervention. We considered this reactivity bias as not substantial, as discussed previously (5).

In conclusion, the current findings suggest that in healthy middle-aged adults, the consumption of equal amounts of coconut oil, olive oil or butter leads to differential changes in plasma FAs, largely driven by differences in the predominant FAs in these dietary fats. There was a marked difference in the effects of coconut oil and butter on individual plasma FA, despite both being major sources of SFAs. Further studies are required to confirm these findings, and to assess potential dietary fat-related changes in the concentration of individual FAs across different plasma lipid fractions in the long term, and in relation to cardiometabolic disease risk.

## Data availability

Data described in the manuscript are available from the corresponding author upon reasonable request.

## Supplemental data

This article contains supplemental data.

## Conflicts of interest

The authors declare that they have no conflicts of interest with the contents of this article.
