# Supplementary material for: Effects of coconut oil, olive oil, and butter on plasma fatty acids and metabolic risk factors: a randomized trial
Source: J Lipid Res. 2024 Oct 28;65(12):100681. doi: 10.1016/j.jlr.2024.100681 (PMC11618001; doi:10.1016/j.jlr.2024.100681)
Supplement: Supporting Materials [file mmc1.pdf]

**Supplemental Information**

# Effects of coconut oil, olive oil, and butter on plasma fatty acids and metabolic risk factors: a randomised trial

Solomon A Sowah[1], Albert Koulman[1], Stephen J Sharp[1], Fumiaki Imamura[1], Kay-Tee Khaw[1]
Nita G Forouhi[1]

[1]Medical Research Council Epidemiology Unit, University of Cambridge School of Clinical Medicine, Cambridge, UK

## Supplemental Tables

**Supplemental Table S1.** The fatty acids composition of the coconut oil, olive oil and butter consumed in the COB trial[a]

| | Common name | Coconut oil | Olive Oil | Butter |
|---|---|---|---|---|
| **Major fatty acids ($\geq$5 mol%)** | | | | |
| C8:0 | Caprylic acid | 8.6 | <0.1 | 1.2 |
| C10:0 | Capric acid | 6.3 | <0.1 | 2.5 |
| C12:0 | Lauric acid | 47.6 | <0.1 | 3.0 |
| C14:0 | Myristic acid | 18.6 | <0.1 | 10.6 |
| C16:0 | Palmitic acid | 8.6 | 14.8 | 28.1 |
| C18:0 | Stearic Acid | 3.4 | 3.0 | 12.4 |
| C18:1n9 | Oleic Acid | 5.2 | 63.5 | 22.2 |
| C18:2n6 | Linoleic Acid | 0.8 | 11.9 | 1.9 |
| **Minor fatty acids (<5 mol%)** | | | | |
| C4:0 | Butyric acid | <1.0 | <0.1 | 2.5 |
| C6:0 | Caproic acid | 0.7 | <0.1 | 1.9 |
| C14:1 | | <0.1 | <0.1 | 0.9 |
| C15:0 | | <0.1 | <0.1 | 1.1 |
| C16:1 | Palmitoleic acid | <0.1 | 1.5 | 1.4 |
| C17:0 | | <0.1 | <0.1 | 0.6 |
| C17:1 | | <0.1 | <0.1 | 0.4 |
| C18:1t | | - | <0.1 | 3.2 |
| C18:1n7 | cis-Vaccenic Acid | <0.1 | 2.8 | 0.4 |
| C18:2tt | | <0.1 | <0.1 | 0.5 |
| C18:2ct | | <0.1 | <0.1 | 0.1 |
| C18:2tc | | <0.1 | <0.1 | 0.2 |
| C18:3n6 | Gamma Linolenic Acid | <0.1 | <0.1 | <0.1 |
| C18:3n3 | Alpha-Linolenic Acid | <0.1 | <0.1 | 0.9 |
| C20:0 | Arachidic acid | <0.1 | <0.1 | 0.2 |
| C20:2n6 | Eicosadienoic acid | <0.1 | <0.1 | <0.1 |
| C18:4n3 | Stearidonic acid | <0.1 | 0.2 | 0.1 |
| C20:1 | Paullinic acid | <0.1 | <0.1 | <0.1 |
| C22:0 | Behenic Acid | <0.1 | 0.2 | 0.1 |
| C22:1n9 | Erucic Acid | <0.1 | <0.1 | 0.1 |
| C22:2 | Docosadienoic acid | <0.1 | 0.6 | <0.1 |
| C24:0 | Lignoceric acid | <0.1 | <0.1 | <0.1 |
| **Fatty acid classes** | | | | |
| Saturated fatty acids | - | 93.8 | 18.0 | 64.2 |
| Monounsaturated fatty acids | - | 5.2 | 67.8 | 28.6 |
| Polyunsaturated fatty acids | - | 0.8 | 12.7 | 3.7 |
| *Trans* fatty acids | - | <0.1 | <0.1 | 4.0 |

[a]The fatty acid composition of the dietary fats used in the intervention was analysed by the United Kingdom Accreditation Service accredited West Yorkshire Analytical Services (Leeds, UK), adopted from the online supplementary appendix 1 (Supplementary File 2) of the authors' previous publication: Khaw KT, Sharp SJ, Finikarides L, et al., Randomised trial of coconut oil, olive oil or butter on blood lipids and other cardiovascular risk factors in healthy men and women, BMJ Open 2018;8:e020167. doi: 10.1136/bmjopen-2017-02016

**Supplemental Table S2. Multivariable-adjusted association between changes in selected individual fatty acids and fatty acid classes with changes in metabolic marker concentrations and C-reactive protein concentration in the COB Trial (n=88)**

| Fatty acid type | Estimates (95% confidence intervals)[a] | | | | | |
|---|---|---|---|---|---|---|
| | HDL-cholesterol | Total cholesterol | Total cholesterol / HDL-cholesterol | Non-HDL-cholesterol | Triglycerides | C-reactive protein |
| | Δ = 0.15±0.26[b] | Δ = 0.23±0.55 | Δ = 0.09±0.39 | Δ = 0.08±0.49 | Δ = 0.01±0.42 | Δ = -0.03±1.16 |
| **Selected fatty acids** | | | | | | |
| Lauric acid, C12:0 | 0.00 (-0.07, 0.07) | -0.07 (-0.23, 0.08) | -0.07 (-0.17, 0.04) | -0.08 (-0.21, 0.05) | -0.02 (-0.14, 0.11) | 0.10 (-0.21, 0.41) |
| Myristic acid, C14:0 | 0.02 (-0.05, 0.10) | 0.12 (-0.04, 0.28) | 0.03 (-0.08, 0.14) | 0.08 (-0.05, 0.22) | 0.01 (-0.11, 0.14) | -0.16 (-0.48, 0.15) |
| Palmitic acid, C16:0 | 0.04 (-0.02, 0.09) | 0.08 (-0.04, 0.19) | 0.01 (-0.07, 0.09) | 0.04 (-0.05, 0.14) | 0.03 (-0.07, 0.12) | 0.17 (-0.09, 0.42) |
| Stearic acid, C18:0 | -0.04 (-0.10, 0.01) | -0.03 (-0.15, 0.09) | 0.01 (-0.07, 0.09) | 0.01 (-0.09, 0.11) | -0.02 (-0.11, 0.07) | -0.15 (-0.41, 0.11) |
| Oleic acid, C18:1n9c | -0.09 (-0.15, -0.04) | -0.13 (-0.26, -0.01) | 0.07 (-0.01, 0.16) | -0.05 (-0.16, 0.05) | 0.13 (0.03, 0.23) | -0.11 (-0.36, 0.14) |
| Linoleic acid, C18:2n6c | -0.02 (-0.07, 0.04) | -0.04 (-0.15, 0.08) | -0.01 (-0.09, 0.07) | -0.03 (-0.13, 0.07) | 0.01 (-0.09, 0.10) | -0.11 (-0.34, 0.12) |
| *Trans* linoleic acid, C18:2n6*t* | -0.06 (-0.11, 0.00) | 0.05 (-0.08, 0.18) | 0.13 (0.05, 0.22) | 0.10 (0.00, 0.21) | 0.04 (-0.07, 0.14) | -0.43 (-0.68, -0.18) |
| **Fatty acid classes** | | | | | | |
| Coconut fatty acids | 0.02 (-0.05, 0.10) | 0.10 (-0.06, 0.26) | 0.02 (-0.09, 0.13) | 0.07 (-0.07, 0.20) | 0.01 (-0.12, 0.14) | -0.14 (-0.46, 0.18) |
| Dairy fatty acids | 0.02 (-0.04, 0.09) | -0.04 (-0.18, 0.10) | -0.04 (-0.13, 0.05) | -0.07 (-0.19, 0.04) | -0.02 (-0.13, 0.09) | 0.16 (-0.12, 0.44) |
| Trans fatty acids | 0.02 (-0.03, 0.08) | 0.05 (-0.07, 0.17) | 0.02 (-0.07, 0.10) | 0.03 (-0.08, 0.13) | 0.02 (-0.08, 0.12) | -0.11 (-0.35, 0.14) |
| MUFAs | -0.09 (-0.14, -0.04) | -0.13 (-0.25, -0.01) | 0.07 (-0.01, 0.15) | -0.05 (-0.15, 0.05) | 0.14 (0.04, 0.23) | -0.08 (-0.33, 0.16) |

[a]A regression model was fitted with an independent variable of observed changes in each fatty acid variable over the follow-up and a dependent variable of changes in each marker. Estimates represent the difference in changes in each marker per 1 standard-deviation change in each fatty acid variable, adjusted for age, sex, baseline body mass index, pre-intervention (baseline) outcome values and randomisation arms. Units were mmol/L for lipids and mg/L for C-reactive protein. HDL, high-density lipoprotein, LDL, low-density lipoprotein.

[b]Mean and standard deviation of changes (post minus pre intervention) of each marker are presented.

**Supplemental Figures**

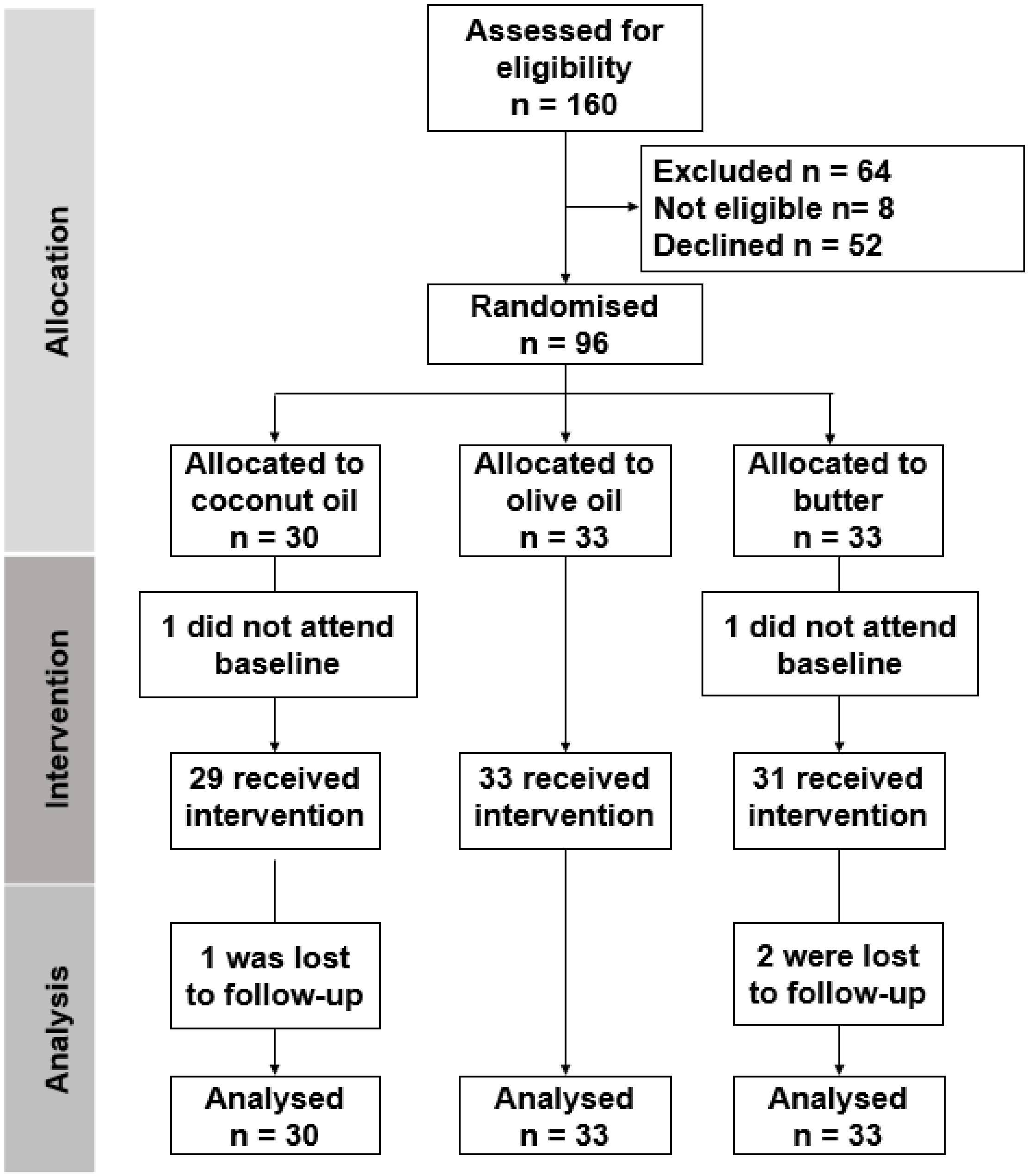


**Supplemental Figure S1.** CONSORT diagram of the COB Trial. CONSORT = consolidated standards of reporting trials. Adapted from Khaw et al. 2018 (1). In the primary analysis, an intention-to-treat approach was undertaken.

**References**

1. Khaw KT, Sharp SJ, Finikarides L, Afzal I, Lentjes M, Luben R, et al. Randomised trial of coconut oil, olive oil or butter on blood lipids and other cardiovascular risk factors in healthy men and women. BMJ Open. 2018;8(3):e020167.
